# Fabrication of Conductive, High Strength and Electromagnetic Interference (EMI) Shielded Green Composites Based on Waste Materials

**DOI:** 10.3390/polym14071289

**Published:** 2022-03-23

**Authors:** Azam Ali, Fiaz Hussain, Muhammad Farrukh Tahir, Majid Ali, Muhammad Zaman Khan, Blanka Tomková, Jiri Militky, Muhammad Tayyab Noman, Musaddaq Azeem

**Affiliations:** 1 Department of Materials and Textile Engineering, Technical University of Liberec, 461 17 Liberec, Czech Republic; zamankhan017@yahoo.com (M.Z.K.); blanka.tomkova@tul.cz (B.T.); jiri.militky@tul.cz (J.M.); tayyab_noman411@yahoo.com (M.T.N.); drmusaddaqazeem@gmail.com (M.A.); 2 Department of Fiber and Textile Technology, University of Agriculture, Faisalabad 38000, Pakistan; 3 Department of Biochemistry, University of Jhang, Jhang 35200, Pakistan; farrukhgcuf@gmail.com; 4 Department of Chemistry, Riphah International University, Faisalabad 46000, Pakistan; majid.ali@riphahfsd.edu.pk

**Keywords:** Kevlar pretreatment, ball milling, carbon particles, conductive composites, thermal conductivity

## Abstract

Conventional conductive homopolymers such as polypyrrole and poly-3,4-ethylenedioxythiophene (PEDOT) have poor mechanical properties, for the solution to this problem, we tried to construct hybrid composites with higher electrical properties coupled with high mechanical strength. For this purpose, Kevlar fibrous waste, conductive carbon particles, and epoxy were used to make the conductive composites. Kevlar waste was used to accomplish the need for economics and to enhance the mechanical properties. At first, Kevlar fibrous waste was converted into a nonwoven web and subjected to different pretreatments (chemical, plasma) to enhance the bonding between fiber-matrix interfaces. Similarly, conductive carbon particles were converted into nanofillers by the action of ball milling to make them homogeneous in size and structure. The size and morphological structures of ball-milled particles were analyzed by Malvern zetasizer and scanning electron microscopy. In the second phase of the study, the conductive paste was made by adding the different concentrations of ball-milled carbon particles into green epoxy. Subsequently, composite samples were fabricated via a combination of prepared conductive pastes and a pretreated Kevlar fibers web. The influence of different concentrations of carbon particles into green epoxy resin for electrical conductivity was studied. Additionally, the electrical conductivity and electromagnetic shielding ability of conductive composites were analyzed. The waveguide method at high frequency (i.e., at 2.45 GHz) was used to investigate the EMI shielding. Furthermore, the joule heating response was studied by measuring the change in temperature at the surface of the conductive composite samples, while applying a different range of voltages. The maximum temperature of 55 °C was observed when the applied voltage was 10 V. Moreover, to estimate the durability and activity in service the ageing performance (mechanical strength and moisture regain) of developed composite samples were also analyzed.

## 1. Introduction

The growing environmental consciousness has forced the research society to place more emphasis on the development of sustainable, green, and environmentally friendly products. In one way, these objectives can be achieved by utilizing suitable natural resources and recycling waste materials [1,2]. The bio-composites (biopolymers reinforced with natural fibers) can be considered as attractive substitutions for non-biodegradable composites [3]. The biocomposites are well known for their degradability into natural products within a short time frame. Currently, research is focused on the fabrication of electrically conductive and EMI shielded composites, by using biopolymers with a combination of conductive fillers [4]. The novel properties of conductive composites such as electromagnetic shielding, electrical conductivity, recyclability, good mechanical behavior, and biodegradability allow for their use in a broad area of applications such as biosensors, bio actuators, fuel cells, drug transfer neural probes, antennas, chemical sensors, etc. [4,5,6]. For instance, in a variety of EMI shielded panels, these composites could prevent the possible explosions of guided aircraft due to electromagnetic interference. The electrical conductivity and EMI shielding ability of such composites mostly depend on the type (metal-based or carbon-based) and physical parameters (size, concentration, structure, processing, and dispersion) of the conductive fillers used. The extent of SE improvement with the gradient increases, creating longer fibers [7,8].

Much research has been carried out to develop and analyze the electrical, dielectric, and EMI shielding effectiveness of conductive polymeric composites [9,10,11]. Zhan et al. explored natural conductive composites based on epoxy/chicken feathers, which have a 2–4 times higher electrical resistivity than glass fiber composites. The study showed good potential to fabricate economical and cost-effective composites but failed to describe the mechanical performance of developed samples, as chicken feathers are less compatible with resin (makes poor interference) [12]. Jia et al. also developed conductive composites using polyaniline filled DBSA and epoxy resin. They found that the fabricated nanocomposites (PANI-DBSA nanocomposites synthesized by an in situ intercalation/polymerization method) showed very good conductivity (10^−3^ S/cm) as compared to the bulk PANI-DBSA at certain contents of resin. However, the PANI has a low specific strength, which leads to segregation while interacting with resin. Chen developed conductive composites using carbon fibers (5 wt% and 5 mm length) and found the minimum electrical resistivity of about 6 × 10^3^ Ω cm. The low thermal stability hinders the use of PVC in some thermal applications. Moreover, these studies were conducted with synthetic resins, and had a poor impact on the environment in terms of degradation [13,14].

The selection of basic constituents (resin and filler) is very important to fabricate the bio conductive composites. Generally, the conductive polymeric composites are developed by using suitable conductive fillers and polypropylene (PP), polyethylene (PE) [15], high-density polyethylene [16], unsaturated polyester [17], and epoxy [18] matrices. These polymeric resins act as barriers to the recyclability of conductive composites. Secondly, due to their molecular structure, these polymers always showed poor mechanical properties. Researchers have been improving the mechanical properties of conductive polymeric composites by creating blends with other fillers [19].

However, green resins are eco-friendly and there are limited problems associated with their recycling [20]. Green epoxy, a plant-based resin, contains 56% bio-renewable molecules [21]. The second most important constituent is the conductive filler. Many studies have reported the use of carbon-based fillers (carbon nanotubes, carbon black, fullerenes graphite and graphene, etc.) for the fabrication of conductive composites. However, the improper dispersion, aggregation, and poor solubility of carbon particles have been challenging issues for researchers [22]. A lack of electrical conductivity and poor compatibility was noticed in previous studies between matrix/resin and carbon fillers. Surface modification/functionalization of carbon-based filler is often required to enhance the fiber (filler) matrix bonding and compatibility. Recently, activated carbon particles have been discussed for the enhancement of electrical conductivity and a fiber-matrix interface [23]. In another study, the thermal and mechanical properties of composites were improved by the reinforcement of bamboo charcoal particles in a combination of polylactic acid resin [24].

To solve the environmental and mechanical (less strength) issues related to electrically conductive composites, this research work focused on the fabrication of conductive composites with the combination of bio-resin, pretreated Kevlar fibers, and activated carbon particles. The Kevlar fibers were pretreated to enhance the uptake of resin during composite fabrication. The activated carbon particles are supposed to provide better bonding (being porous) and good electrical properties, with additional advantages of inexpensive filler and less toxicity. The activated particles were subjected to the ball milling process to convert them into fine scale. Then, various concentrations of these particles were embedded in green epoxy to develop the electrically conductive composites. The conductive composites were tested for electrical, mechanical, and thermal properties.

## 2. Materials and Methods

The Kevlar woven fabric wastes were obtained from VEBA textiles, Hradec Králové, Czech Republic. The Green epoxy resin CHS-Epoxy G520 and hardener TELALIT 0600 were supplied by Spolchemie, nad Labem, Czech Republic. The rest of the auxiliaries Methanol (99.8%) and Calcium carbonate (≥99%) were obtained from Sigma Aldrich. The activated carbon particles were obtained from Anusawari, Bang Khen, Bangkok and Thailand. The 3-(Trimethoxysilyl)propyl methacrylate (Catalog Number M6514) and n-Propyltriethoxysilane were obtained using Merck chemicals, Burlington, MA, USA.

### 2.1. Methodology

#### 2.1.1. Preparation of Kevlar Web

**Figure 1:**
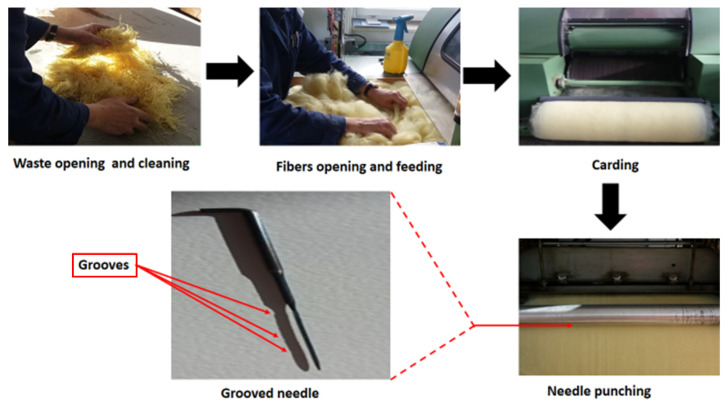
Schematic diagrams to convert Kevlar waste into a nonwoven web.

Kevlar waste was obtained from the industry. The waste contained fabric pieces, yarns, and fibers. Subsequently, waste was subjected to opening, cleaning, garneting, and carding to form a web. The web was then transferred to a needle-punched non-woven machine. The frame of the needle had a density of 48 needles/cm^2^ and the speed was 0.5 m/min. The complete methodology to convert Kevlar waste into the nonwoven web is shown in Figure 1.

#### 2.1.2. Chemical Treatment of Kevlar Nonwoven Web

Surface cleaning was carried out before the pre-treatment of Kevlar fibers. The fabric was immersed in acetone for 5 h then thoroughly washed. Then, the washed fabric was immersed in 5 g/L NaOH solution at 50 °C for 40 min to enhance the bonding tendency of fibers. Solution 2 was made by adding 1 wt% 3-(Trimethoxysilyl)propyl methacrylate and 1 wt% n-Propyltriethoxysilane in distilled water at an acidic pH (by adding a few drops of acetic acid). The coupling agents were dissolved with the help of a magnetic stirrer until 3-(Trimethoxysilyl)propyl methacrylate turned transparent. Subsequently, the pretreated Kevlar was immersed in solution 2 for 20 min at 25 °C. Then, the treated fibers were dried out at 120 °C for 90 min in the oven.

#### 2.1.3. Preparation of Particles

At first, the activated carbon particles were converted into small particles based on previous research experience [25]. The high-energy planetary ball milling (Fritsch pulverisette 7, Weierbach, Germany) was utilized for dry pulverization. The sintered corundum container (80 mL) and zirconium balls (10 mm dia.) were used for the dry milling of the sample. The time period of 60 min was selected for dry milling. The ball milling processing parameters, of time, ball-to-material ratio (BMR), and speed were 60 min, 10:1, and 850 rpm.

#### 2.1.4. Preparation of Conductive Paste

At first, 500 mL of methanol was transferred into the beaker and 10 g of calcium carbonate (CaCO_3_) was soaked in the beaker overnight. The purpose of soaking the CaCO_3_ into methanol was to eliminate the water present in methanol. Subsequently, CaCO_3_ was removed and the whole solution was poured into a bottle. A total of 100 mL of solution was taken into a separate beaker and 1 g of activated carbon particles was dispersed in it using an ultrasonic probe disperser at 30 °C for 30 min. It was termed as solution 1.

The conductive paste was made by adding100 mL of green epoxy into solution 1. The solution was constantly stirred for 2 h. Subsequently, the beaker was placed in the oven for 50 min to evaporate the methanol solution. The achieved paste was stirred mechanically for 10 min and resistivity was checked. Hence, three more conductive pastes were made by using 4, 6, and 8 g of the conductive particles.

#### 2.1.5. Fabrication of Composites from the Conductive Paste

The hand layup technique was used to develop the conductive composites. This technique is economical, simple, and very fast as compared to other composite manufacturing techniques. The adopted hand lay-up process was divided into the following four simple steps: mold preparation, gel coating, lay-up, curing of the fabricated composites. A pigmented gel coat was applied onto the mold surface which resulted in ease of handling and a high-quality composite surface. This technique is beneficial to fully impregnate the reinforcement (fabric) by applying the pressure with the brush. Then, hand rollers (epoxy laminating grooved rollers) were used to ensure an enhanced interaction between the fibers and the polymeric resin. Subsequently, wet composites were pressed with a hand roller to ensure an enhanced interaction between fibrous reinforcement and epoxy resin, and uniform resin distribution free from air bubbles or voids. In the final step, the developed composites were cured under the standard atmospheric conditions, where hardening of the composites was obtained without applying external heat. The skills to laminate the fibrous reinforcement and epoxy resin play a pivotal role in the quality and performance characteristics of the fabricated composites developed by using the hand lay-up technique.

The recommended hardener was mixed in a previously made conductive paste at a ratio of 100:20 (by weight). For the fabrication of composite, the mould release agent (wax or oil) was applied over the surface of the mould. A small amount of resin was poured over the surface and spread with a brush. Then, Kevlar fabric was placed over the applied resin and squeezed with the brush. Hence, the resin was continuously added and squeezed with the brush, followed by each layer of reinforcement. Therefore, five different samples of the composite were made. Four samples CH1, CH2, CH3, and CH4 were made with four previously made conductive pastes (having 2, 4, 6, and 8 g of carbon particles) and one C0 sample was made with a simple resin with no carbon particles. The design of the experiments for the composite samples is shown in Table 1.

**Figure 2:**
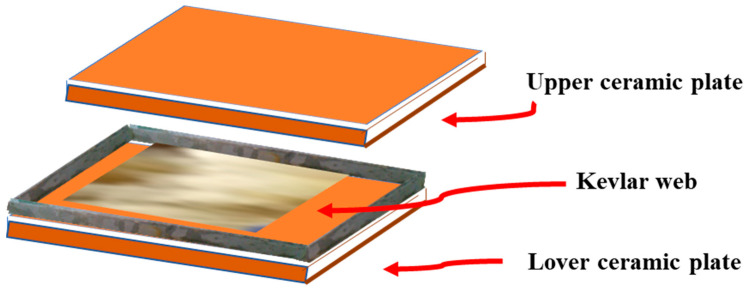
Schematic for mould and fabrication of composites.

At first, all the composite samples were cured at room temperature for 24 h. The post-curing was performed in an oven at 110 °C for 4 h. The problems arising during the hand layup technique are unevenness, non-uniformity in thickness, and improper dimensions. All these problems were capped via the design of a special mould. Two ceramic plates were used. The lower plate was fixed with marble edges with a height of 3 mm. It is the beauty of this mould that the fibers are properly impregnated by applying a certain pressure up to the required thickness level. The schematic of the designed mould is given below in Figure 2.

## 3. Characterizations

### 3.1. Surface Morphology Testing

The surface morphology of the developed activated carbon web, particles, and conductive composites was analyzed using a scanning electron microscope (SEM). SEM (Tescan VEGA III TS5130, Brno, Czech Republic) was used at an accelerated voltage of 10 kV. The size of carbon particles after milling was analyzed by the particle size distribution method. A Malvern zetasizer based on the principle of dynamic light scattering of the Brownian motion of activated carbon particles was used. The dispersion of activated carbon particles was made using the recommended solvent and sonicated before characterization. Fourier transform infrared spectroscopy (FTIR) was used to study the developed functional groups. The ATR-FTIR (Nicolet iZ10, Thermo Scientific Inc., Waltham, MA, USA) analysis for untreated and chemically treated Kevlar fibers was analyzed in the wavelength range of 600–4000 cm^−1^.

### 3.2. Electrical Resistivity

**Figure 3:**
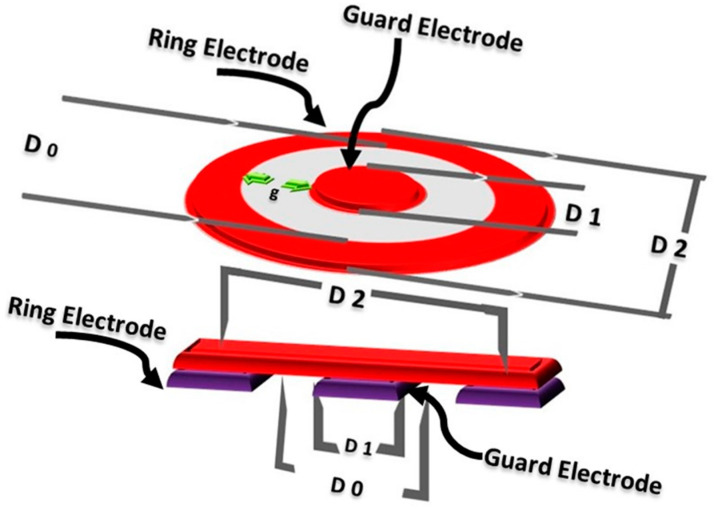
Scheme of concentric electrodes for the measurement of electrical resistance.

The standard ASTM D257-07 was followed to measure the electrical resistivity. It was measured using the concentric electrodes. The direct current supply was maintained at 100 V under the constant pressure of 2.3 kPa. All the conductive samples were kept under standard conditions (21 °C and the relative humidity 54%) for 24 h. The test was also performed in the same laboratory under standard conditions. The sample was placed between two circular electrodes and a voltage supply was maintained to obtain the resistance. Hence, the volume resistivity *ρ_v_* (Ω mm) was calculated by using Equation (1)

(1) $$\rho_{v}=R_{v}\left(\frac{S}{t}\right)$$

where *R_v_* (Ω) is the reading of volume resistance, *t* is the thickness of the sample (mm), *S* is the surface area of the electrode (mm^2^) (π*D*_2_^2^/4), *D*_2_ is the inner diameter of the outer ring electrode (mm) [26]. *D*_0_ is (*D*_2_ − *D*_1_)/2, *D*_1_ is the outer diameter of the center electrode (50.4 mm), *D*_2_ is the inner diameter of the outer ring electrode (69 mm) and *g* is the distance between *D*_1_ and *D*_2_. The Figure 3 is showing the scheme of concentric electrodes for the measurement of electrical resistance

Five readings of each composite sample were taken at different places and the average value was calculated. The error bars are the standard deviation.

### 3.3. Electromagnetic Shielding Testing

Electromagnetic shielding testing effectiveness was measured using two different methods. The first method (ASTM D4935-10) is based on the coaxial transmission insertion loss principle. The measuring range of frequency lies between 30 MHz to 1.5 GHz. The measurement assembly consisted of an input connector, sample holder, and the output connected to the network analyzer. A shielding effectiveness test fixture (Electro-Metrics, Inc., model EM-2107A, Johnstown, NY, USA) was used to hold the sample. A network analyzer (Rohde & Schwarz ZN3, Munich, Germany) was used to generate electromagnetic radiations.

**Figure 4:**
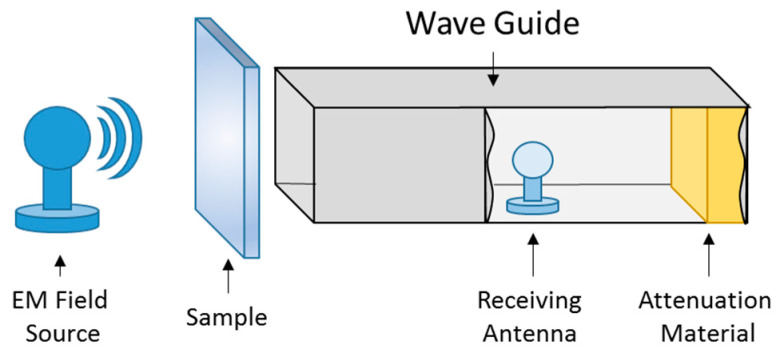
Schematic diagram of waveguide method set up.

The second method is called the waveguide method. The working principle resembles ASTM D4953-10 but is slightly different. This method provides the Electromagnetic shielding effectiveness in a broad range of frequencies up to 2.45 GHz. The waveguide method is most suitable for characterizing the EMI shielding of conductive composites [27]. The main part of the device is a hollow waveguide. This waveguide is made up of electrically conductive metals. The sample was placed at the entrance of the conductive waveguide and the receiver part was placed inside. EM signals were generated by a network analyzer Agilent E 4991A and a high-frequency analyzer HF-38B (Gigahertz Solutions, Langenzenn, Germany) was used to receive them. The setup is shown in Figure 4.

The ratio between transmitted to incident power of the electromagnetic waves was calculated to express the effectiveness of EMI shielding (*SE*) in *dB* as depicted in Equation (2).

(2) $$SE\;\left(dB\right)=10\;log\frac{P_{t}}{P_{i}}$$

where *P_t_* and *P_i_* are the power density (W/m^2^) measured in presence of sample (transmitted), and without the sample (incident), respectively.

Each sample was tested three times at different places and the average value was calculated. The error bars are the standard deviation.

### 3.4. Characterization of Mechanical Properties

**Figure 5:**
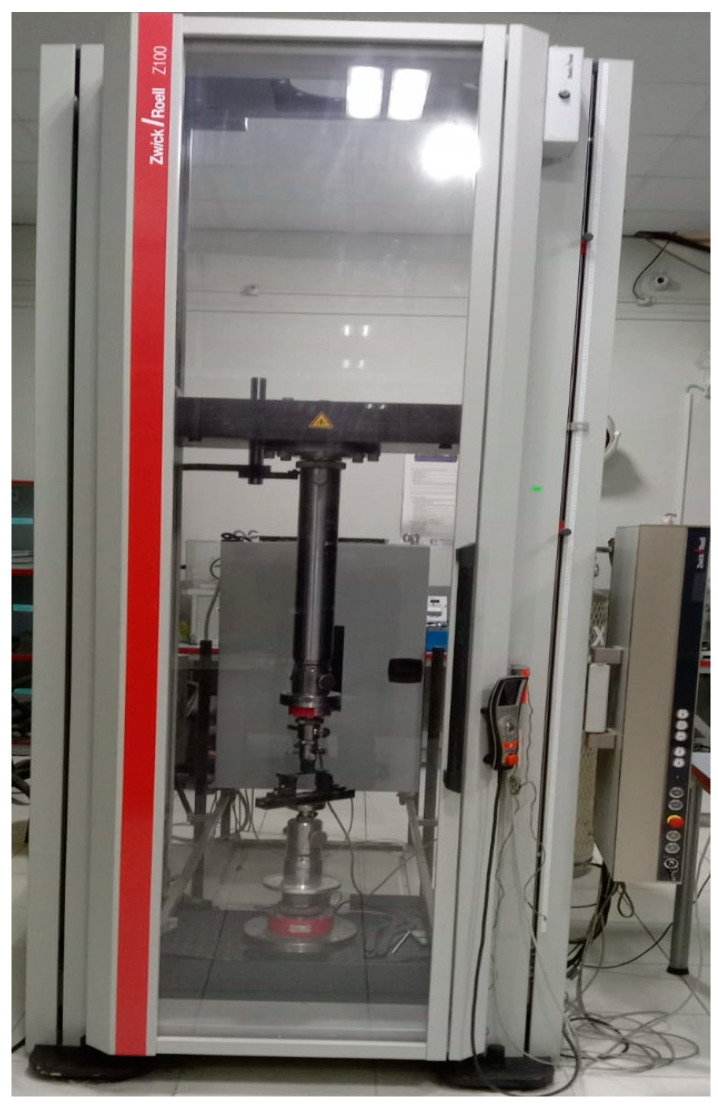
UTM used for the analysis of mechanical properties of the developed composites.

The mechanical properties were tested for all of the developed composite samples. A Universal Testing Machine (UTM) (Zwick/Roell Z100, Ulm, Germany) with a load cell capacity of 100 KN was used to measure the mechanical properties of the developed specimens as shown in Figure 5. The ultimate tensile strength was determined by the universal tensile strength tester according to the standard test method (ASTM D3039, West Conshohocken, PA, USA). Samples were cut into flat strips with rectangular cross-sections according to the recommendation of the standard.

The prepared samples were fixed in the jaws of the testing machine. The machine operates with the working principle of a constant rate of loading with a fixed speed, in which the applied load changes from zero to breaking load. The breaking load is the measure of the mechanical resistance of the developed composites [28]. The stress–strain (SS) behavior of the fabricated composites was recorded and analyzed. The ultimate tensile strength, ultimate tensile strain, and tensile modulus were obtained from the SS curve and analyzed.

The standard test method, ASTM D7264, was used to analyze the flexural strength of the developed electrically conductive composites using a three-point bending tester. For this, specimens with a standard span-to-thickness ratio (20:1), width (13 mm) length (20% longer than the support span) were prepared as per standard [29].

Each composite sample was tested three times and the average value was calculated. The error bars are the standard deviation

### 3.5. Heating Performance of Conductive Fabrics

The ohmic heat was measured for all fabricated composite samples. The composite was clamped in silver-coated electrodes The range of different voltages was applied for different time intervals. The amount of heat generated is related to I^2^ as given in Equation (3).

P = I^2^·R (3)

where P denotes the total power dissipation, R denotes the resistance of the operating heater and I denotes the current passing through conductive samples. At 5 Volt with a time interval of the 1 min, the temperature of conductive yarns was noted and images were captured with an FLIR thermo-camera.

### 3.6. Thermal Conductivity

The electrically conductive materials always showed thermal conductivity in behavior. The thermal conductivity of electrically conductive developed composite samples was measured using an Alambeta measuring device (Sensora Instruments, Liberec, Czech Republic. The instrument consists of two plates and a sample is always placed between these plates. The heat is generated on the upper plate and passes through the sample by convection. The following equation was used to calculate the thermal conductivity.

R = h/λ(4)

where R is the thermal resistance of the samples (W^−1^ km^2^, h is fabric thickness (m) and λ is thermal conductivity W (m K)^−1^. Each composite sample was tested three times and the average value was calculated. The error bars are the standard deviation.

## 4. Results and Discussion

### 4.1. Particle Size Analysis

**Figure 6:**
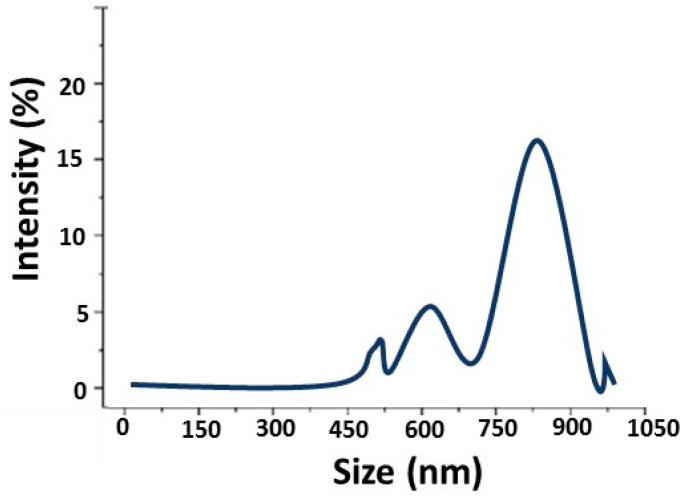
Size distribution of dry milled (60 min) carbon particles.

The activated microparticles were used as a conductive material in the present research. The particles were subjected to dry milling before use in composites. The machine consisted of marble balls and operated at the multimodal distribution of dry milling. The results for particle size and their distribution average are shown in Figure 6. The milling process was stopped after every 20 min because further milling results in an increased temperature of the ball mill and subsequent cold welding of carbon particles on the milling container. The average particles size of the developed carbon nanoparticles was determined by the Malvern zetasizer using the dynamic light scattering principle of Brownian motion of particles. The dispersion of activated carbon particles was made in the recommended solvent and sonicated for 20 min with a bandelin ultrasonic probe prior to characterization. The rate of dry milling has a significant effect on the fineness of particles. The size was reduced faster during the initial 20 min, after which it slowly reduced in the next two halves of the following 20 min. The average particle size after 60 min of dry milling was analyzed at about 820 nm.

**Figure 7:**
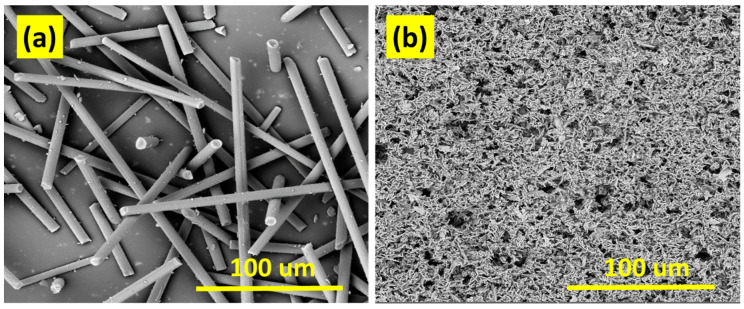
SEM micrographs of carbon fibers/particles, (**a**) Before milling (**b**) After milling.

Furthermore, scanning electron microscopy was employed at each level. It was used to study the morphology of particles, the morphology of Kevlar fibers after chemical treatment, and to study the morphology of composite samples. Additionally, 60 min of dry milling was found to be very effective in changing the shape of particles from micro to nano level. Instead of nano-segments, some of the particles were also found to have been changed to a micro-level (higher aspect ratio) due to shorter milling action. To develop further refined particles, it is vital to pulverize the developed carbon particles for a long time at the controlled temperature of the ball mill. The scanning electron images of particles before and after milling action are shown in Figure 7.

**Figure 8:**
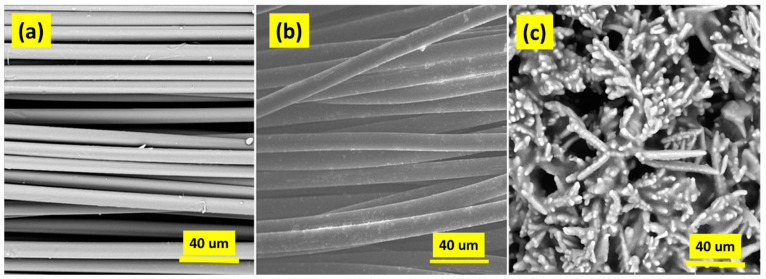
SEM images of (**a**) untreated fiber, (**b**) chemically treated fiber, and (**c**) fiber-reinforced composite.

In the second step, electron microscopy was assessed for Kevlar fibers before and after treatment with chemicals. The surface of fibers was analyzed in depth. Figure 8 shows the noticeable roughness on the surface of the activated fiber as compared to simple untreated Kevlar fiber. The behavior is attributed to the formation of a porous structure on the fibrous structure. Furthermore, the chemical treatment may affect the compact chains of Kevlar and produce some functional groups as shown in the FTIR results.

### 4.2. FTIR Analysis

**Figure 9:**
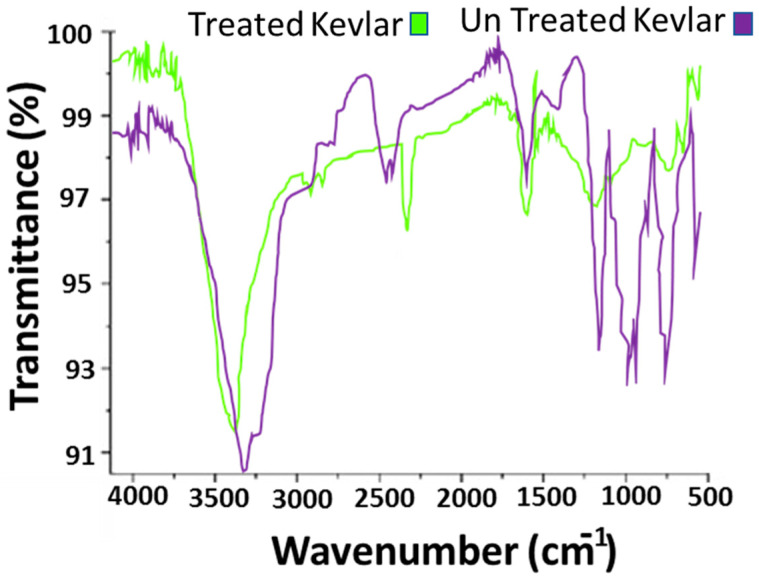
FTIR analysis of Kevlar surface before and after chemical treatment.

The *γ* -methacryloxypropyltrimethoxysilane (*γ* -MPS)/propyltriethoxysilane (*γ* -APS) provide C=O groups, which are responsible for the appearance of two new peaks at 1010 and 3311 cm^−1^ due to the –OH and Si–OH stretching in silane treatment. Due to the pretreatment of NaOH, a strong peak of C=O appeared at 1719 cm^−1^. Furthermore, the alkaline treatment provides free bridging –OH at 3742 cm^−1^. The silane treatment slightly shifted the –OH peak to 3313 cm^−1^ as compared to untreated Kevlar fiber [30]. The FTIR spectra of Kevlar surface before and after chemical treatment is showing in Figure 9.

### 4.3. Electrical Conductivity

#### 4.3.1. Electrical Resistivity of Methanol Solution

**Figure 10:**
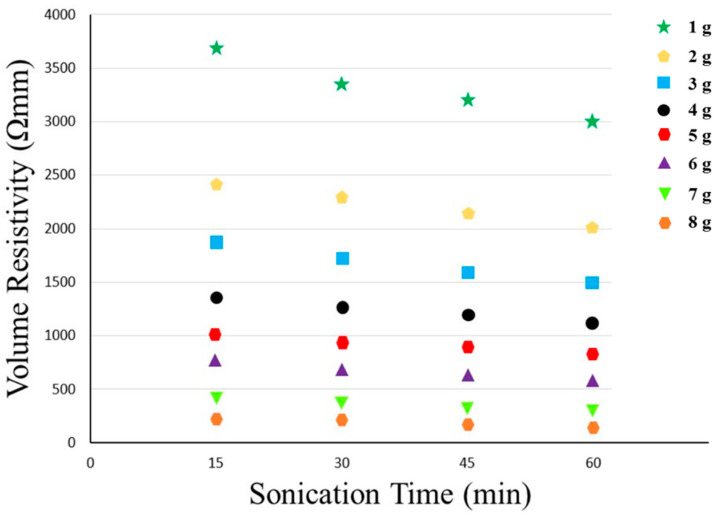
Effect of different concentrations of carbon particles and sonication time on electrical resistivity.

As discussed earlier in Section 2.1.4, 1 g of carbon particles was added to the methanol solution under constant stirring. Then, we placed the whole assembly in a sonicator bath. The resistivity was recorded after 15 min. At that time, we added more conductive particles and the sonication time was increased. Hence, we prepared four samples with 1 g of carbon particles with a sonication interval of 15, 30, 45 and 60 min. Similarly, the resistivity was recorded at different intervals of sonication (15, 30, 45 and 60 min) for 2, 3, 4, 5, 6, 7 and 8 g of carbon particles. In all of the experiments, the amount of methanol was kept at 100 mL. The results of electrical resistivity with a different carbon particle concentration (1 to 8 g) and sonication time are provided in Figure 10.

With 1 g of conductive particles, the values of measured resistivity along 15, 30, 45 and 60 min of sonication decreased due to the proper dispersion of conductive fillers. However, the overall resistivity was still high and therefore, the amount of conductive fillers was increased to 2 g, which significantly reduced the electrical resistivity. The stable and constant values for the electrical resistivity were achieved after sonicating for 60 min. An increase in the concentration of fillers to 3, 4, 5, 6 and 8 g resulted in a noticeable decrease in resistivity. The conductivity improved with an increasing time of sonication. Hence, the lowest resistivity 1098 Ωmm was achieved with 8 g of fillers (particles) sonicated for 60 min.

#### 4.3.2. Electrical Resistivity of Conductive Paste and Composites

**Figure 11:**
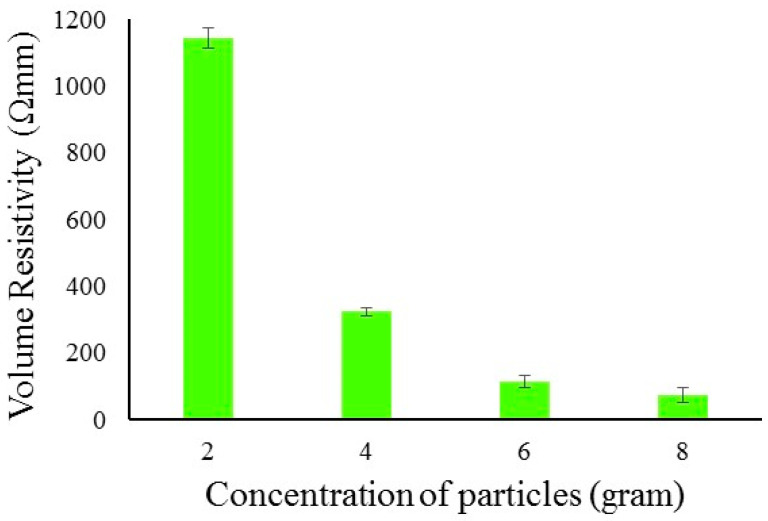
Electrical resistivity of conductive paste.

The composites samples were made by using the conductive solutions with 2, 4, 6 and 8 g of carbon particles sonicated for 60 min. A total of 100 mL of bio epoxy resin was added to each solution and sonicated for 2 h. Almost all of the methanol solution evaporated during the mixing of epoxy and sonication for a long duration of two hours. The addition of epoxy, evaporation of methanol, and sonication (2 h) resulted in an improved resistivity of the solution. This behavior can be attributed to the low viscosity of the particles in pure methanol, and particles aggregate soon after the sonication process. When the viscosity was increased by the addition of epoxy, the dispersion of particles increased due to increased drag on them. This results in a better and more stable dispersion of the particles even after the sonication process [31]. The results of electrical resistivity of conductive paste is showing in Figure 11.

**Figure 12:**
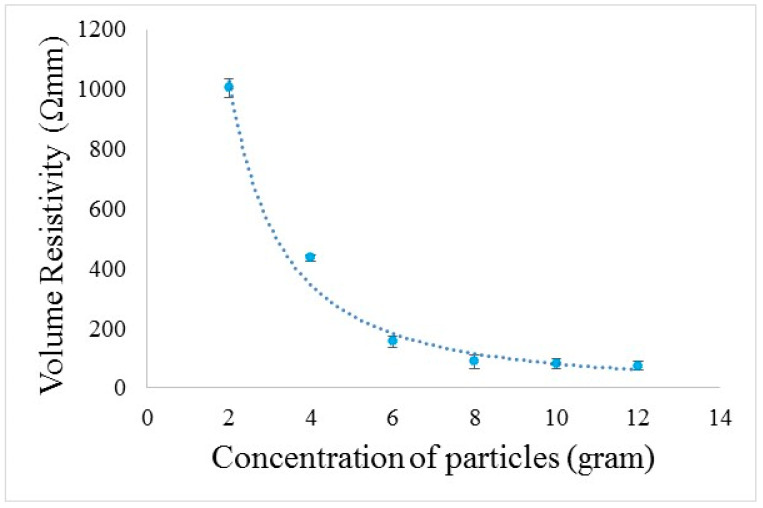

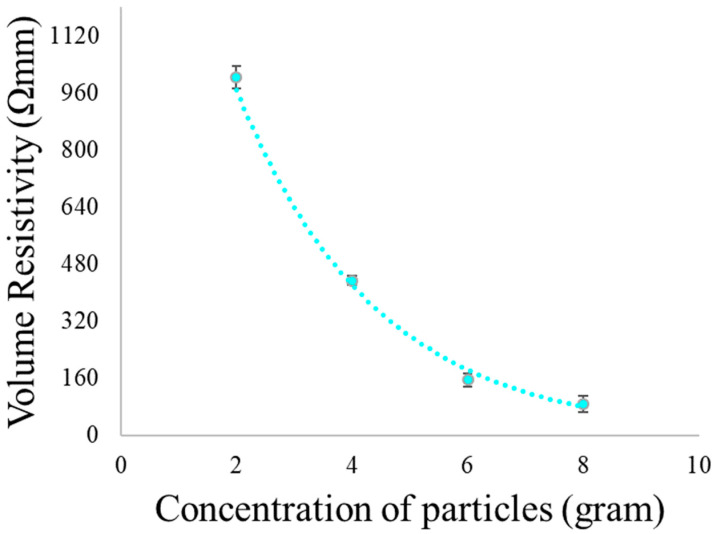
The conductivity of the developed composites.

This conductive paste was used to develop the conductive composites. Therefore, we developed a total of five conductive composite samples. The four samples were of 2, 4, 6 and 8 g of carbon particles and one sample did not have conductive fillers. The electrical resistivity results for all fabricated composite samples are shown in Figure 12. It is clear from the trend line of electrical resistivity that an increase in the concentration of carbon particles has a direct impact, leading to a decrease in electrical volume resistivity (increase in electrical conductivity). Composite samples with 2 g of carbon fillers had an electrical resistivity of about 1000 Ωmm as compared to the lowest resistivity at 90 Ωmm with 8 g of carbon particles. This means the concentration of conductive fillers has a significant effect on an increase in electrical conductivity.

Remarkably, it has been described in the literature that different parameters (metallic impurities and defects) have a diverse effect on the resistivity of conductive materials. Researchers have used a higher concentration of fillers to achieve the percolated threshold (more than 12%) as compared to the present study (achieved threshold with 8% of particles). Moreover, in the present study, green epoxy is used (the novelty of work is considerable regarding resin), while in the literature polyester, styrene, phenolic and epoxy resins have been widely used [32].

Hence, the above-mentioned low concentrations (6 and 8% of fillers) are quite suitable for the percolation threshold to maintain the interconnected network of carbon nanoparticles. The trend line in the graph shows the linear relationship between the electrical resistivity and concentration of particles up to 6%. For the sake of reaching the declining value of electrical resistivity against an increasing concentration of particles, we also developed composite samples with 8, 10 and 12% of carbon particles. Furthermore, it was observed that an electrical threshold of up to 6% led to low resistivity values, but at 8% of particles, there was a further significant decrease in resistivity. However, following a further increase in the concentration of particles to 10% and 12%, there was an insignificant decrease in electrical resistivity as compared to 8% of particles. So, it was decided that 8% of particles was sufficient to provide minimum electrical resistivity. It is also reported that a very high concentration of aggregates corresponds to high percolation thresholds. However, when the amount of fillers is higher than a critical value, the aggregates have almost no influence on the percolation threshold of the composites. Generally, the influence of the aggregates on the performance properties of the fabricated composites is directly influenced by their type [26]. So, it is important to select a proper dispersion process to avoid possible highly concentrated aggregates of CNPs while preparing composites [27]. It is important to keep in mind that our carbon nanoparticles have a higher value of aspect ratio (because of rod-like structures as shown in Figure 6) in comparison to round ball-type particles. It means that by increasing the amount of CNPs, a mechanism of percolation is activated, in which an interconnected network is formed. It is important to note that the overall conductivity of the fabricated composites is better than has been reported in the literature, as Ni-coated short carbon fibers epoxy composites showed a maximum electrical conductivity of 2.1 Ω/mm^2^ [28]. The biocomposites of polylactic acid and carbon fillers (8%) exhibited an electrical conductivity of 2.2 × 10^−5^ S/cm, which is less than our findings for fabricated composites (C6 1.0 × 10^−2^) [29].

### 4.4. Electromagnetic Shielding of Fabricated Conductive Composites

To broaden the applications of conductive composites, the embedding of activated carbon particles into green resin could be considered as a smart option. This includes electromagnetic shielding for doors, panels, and packaging materials of electronic devices where electrostatic charge dissipation is necessary for the safety of electronics [33]. The electromagnetic shielding effectiveness was studied using two approaches (i.e., coaxial transition measurement waveguide principle).

#### 4.4.1. Coaxial Transition Principle

The shielding effectiveness (*SE*) is defined as the ratio of transmitted power (*Pt*) to incident power (*Pi*), and it is measured in decibels (*dB*) using Equation (5).

(5) $$SE\;\left(in\;dB\right)=10log\frac{Pt\;}{Pi}$$

**Figure 13:**
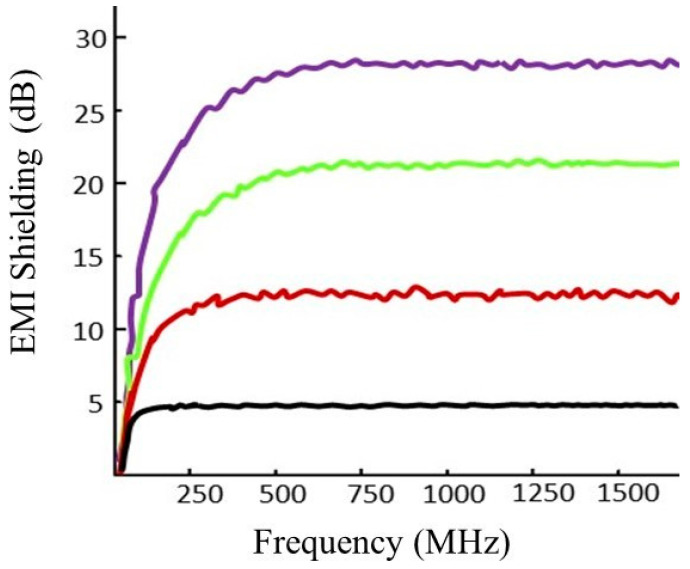
EMI shielding effectiveness of electrically conductive composites.

The shielding effectiveness (*SE*) of all composite samples was measured in the frequency range of 30–1500 MHz. Figure 13 shows the EMI shielding effectiveness for all composite samples. The composite samples were made using the conductive solutions with 2, 4, 6 and 8 g of carbon particles sonicated for 60 min. After the fabrication of composites, the effect of different concentrations of conductive fillers (carbon particles) was observed against the electrical behavior and EMI shielding. The increase in the concentration of conductive fillers demonstrated a significant effect on electromagnetic shielding. The results indicated that EMI shielding increases with an increasing concentration of carbon fillers. The composites fabricated with 8 g of particles showed a maximum of 28.5 dB of EMI shielding as compared to 2 g of carbon particles (4.9 dB). The behavior is attributed to a higher concentration, and a more percolated network showed higher conductivity as compared to 2, 4 and 6 g of carbon particles. Moreover, the electrical conductivity in conductive composites is a result of the migration of electrons (presence of π-cloud) in carbon particles and their jumping across the defect interfaces between discontinuous carbon links [34].

#### 4.4.2. Waveguide Method

**Figure 14:**
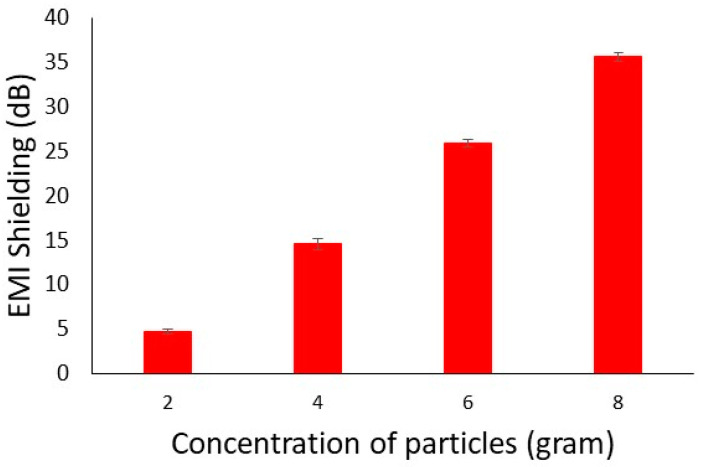
EMI SE of Conductive composites at higher frequency (2.45 GHz).

The waveguide instrument has a broad range of frequencies as compared to the co-axial transmission principle. Hence, all composite samples were tested at a higher frequency of 2.45 GHz. The results of electromagnetic shielding effectiveness of electrically conductive composites at a higher frequency are shown in Figure 14. The EMI shielding is higher for higher concentrations of carbon particles. A higher concentration provides a better threshold for electrical conductivity, which in turn enhances the decibel values of EMI shielding. It is also obvious that EMI shielding values were significantly increased for all composite samples at a higher frequency (2.45 GHz) as compared to the lower frequency provided by the co-axial transmission principle (1.5 GHz).

The electrical conductivity and electromagnetic shielding of the fabricated composites were very good. It was also found that the noticeable changes and EMI SE were observed with the increasing content of carbon fillers. Both the concentration of carbon content and proper mixing of the solution play a vital role in the fabrication of conductive networks with high EMI SE. EMI shielding is significantly improved with increasing frequency.

**Figure 15:**
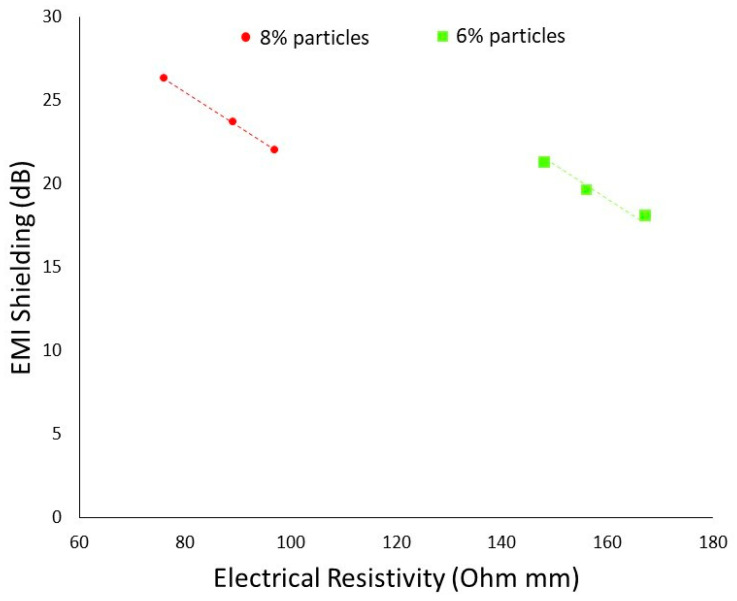
The comparison between electrical conductivity and EMI shielding for composite samples made by 6 and 8 percentage of carbon particles.

Furthermore, a comparative study was performed between electrical conductivity and EMI shielding for two different composite samples containing a different content of carbon fillers (6%, 8%) as shown in Figure 15. As discussed earlier, there is a linear relationship between electromagnetic interference (EMI), shielding effectiveness and electrical conductivity. At a lower value of electrical resistivity (Higher electrical conductivity), the composite samples show higher EMI shielding. The maximum EMI shielding values (27 dB) were achieved, corresponding to a lower resistivity (77 Ωmm).

### 4.5. Heating Performance

The heating of conductive composites was performed by applying the voltage difference across its ends. An increase in the temperature of the surface was observed at different voltages at different intervals of time. At first, five voltages were applied for 1 min and the observed temperature for the conductive composite was around 45.4 °C. In the second step, the time was prolonged up to 10 min at a fixed voltage of 5 V and the surface temperature across the conductive composite increased up to 52 °C. Likewise, in the third step, we increased the voltages (10 V) for 10 min, and the corresponding surface temperature was found to be 94 °C. During the process of ohmic heating, the charges receive flow due to an acceleration of the applied electric potential. Heat is generated once these charge carriers migrate and collide elastically with phonons and defects present in the conductive materials. This phenomenon of joule heating was attributed to an increase in the charge carriers by an increase in voltages.

### 4.6. Thermal Conductivity

**Figure 16:**
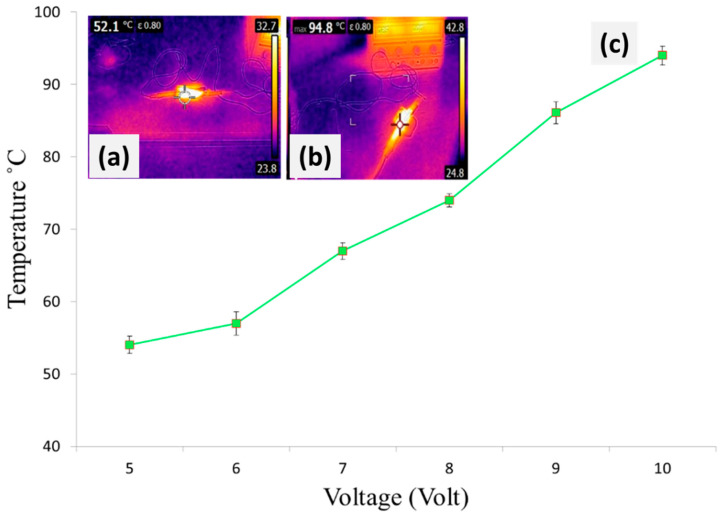
Surface temperature of (**a**) Silver plated fabric at 5 volts and 1 min, (**b**) Silver plated fabric at 5 volt and 10 min, and (**c**) Silver plated fabric at 5–10 volt.

**Figure 17:**
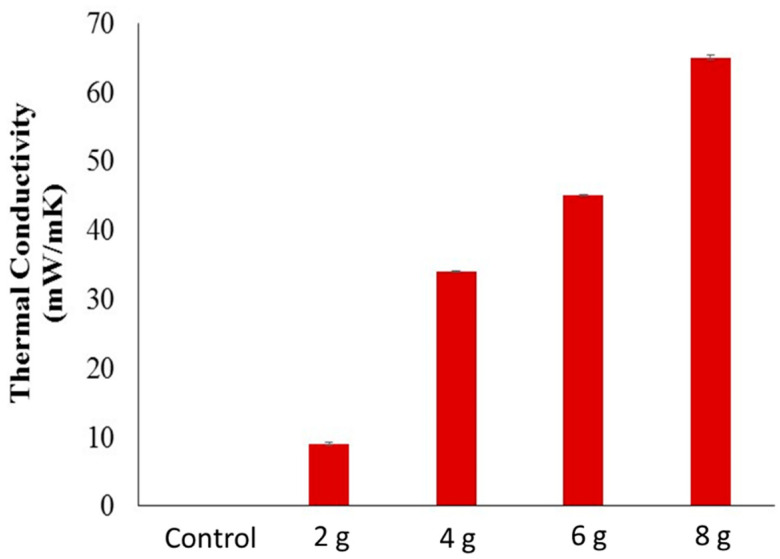
Thermal conductivity of developed composite samples.

The electrically conductive materials always showed thermal conductivity in behavior. The thermal conductivity of the electrically conductive developed composite samples was measured using the Alambeta measuring device (Sensora Instruments) [35]. The instrument consists of two plates and a sample is always placed between these plates. Heat is transferred using a specific area of composite across the thickness. The change in the temperature difference (temperature gradient) was also measured. The results of thermal conductivity are shown in Figure 16. The thermal conductivity of composite sample with no carbon particles (control) is at the very least about 0.05 mW/m/K; however it increased when adding conductive carbon particles in resin. The most conductive composite sample showed a higher thermal conductivity of about 65 mW/m/K. This behavior is attributed to the fact that composite samples have good electrical conductivity. The thermal conductivity depends on tight bonding, vibration, and collision between the molecules. This effect is reinforced if the material also shows electrical conductivity. The presence of a π bond in carbon-based materials makes sure the overlapping of valance band to the conduction band. Hence, the composite samples showed higher thermal conductivity. Thermal conductivity of developed composite samples is showing in Figure 17.

### 4.7. Mechanical Testing

**Figure 18:**
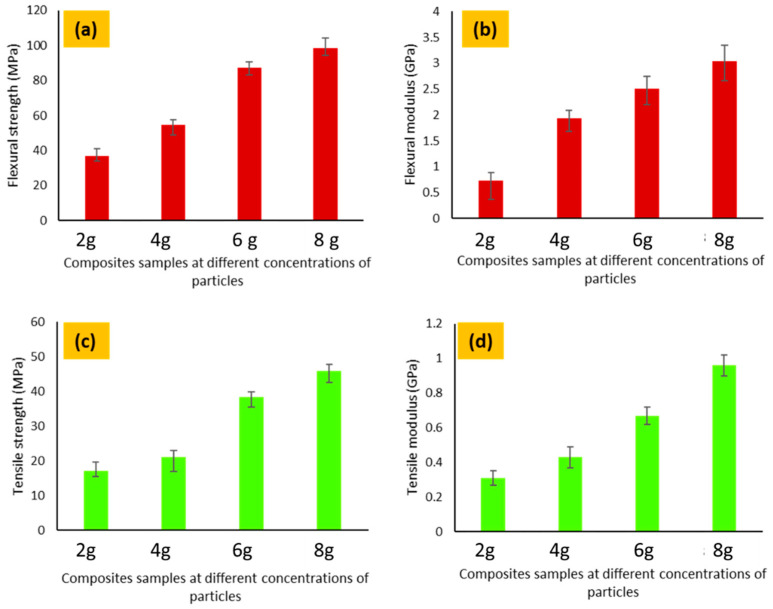
Mechanical properties of conductive composites (**a**) Tensile strength (**b**) Tensile modulus (**c**) Flexural strength and (**d**) Flexural modulus.

The mechanical properties were tested for all of the developed composite samples. The strength at break was determined by the tensile strength tester according to the standard (ASTM D3039). The ultimate tensile strength of fabricated conductive composites was recorded according to the maximum load that composites withstand before failure. The tensile strength of composite samples made with 2, 4, 6 and 8 g of carbon particles is shown in Figure 18. It is clear from the bar graphs of tensile strength that an increase in the concentration of carbon particles has a direct impact on an increase in tensile strength and tensile modulus. Composite samples with 2 g of carbon fillers show the lowest mechanical strength as compared to 8 g of carbon particles. It means the concentration of conductive fillers has a significant effect on an increase in mechanical properties. Remarkably, it is specified in the literature that different parameters (metallic impurities and defects) have a diverse effect on the resistivity of conductive materials. Researchers have used a higher concentration of fillers to achieve the percolated threshold (more than 12%) as compared to the present study (achieved threshold with 8% of particles). Moreover, in the present study, green epoxy was used (the novelty of work is considerable regarding resin), while in the literature polyester, styrene, phenolic and epoxy resins were widely used. It is maybe due to the fact that a higher concentration of particles provides extra support to reinforcements. Furthermore, the mechanical behavior of the composites is directly influenced by the concentration and proper distribution of fillers. It is obvious from the results that the strength increased with an increasing concentration and even distribution of carbon fillers. The adhesion between the matrix and reinforcement significantly improved after the curing process, which may result in increased tensile strength.

The flexural strength of electrically conductive composites developed was measured using a three-point bending tester following the standard test method (ASTM D7264). The results of flexural strength resemble the tensile strength. It can be seen from the results that the bending force in the fabricated composite specimens increased with an increasing concentration of carbon fillers. This indicated that the bending force is also directly influenced by the concentration and even distribution of reinforced carbon fillers. The sample made of 8 g of particles has more flexural strength than all other samples. This behavior can be attributed to the increased concentration and even distribution of carbon fillers in resin, which provides a strong and even network with Kevlar reinforcement. The mechanical properties of the fabricated composites are close to the electrically conductive polylactic acid/carbon fiber composites made by Chien et al. [36] and are considerably higher than the Ni-coated short carbon fiber epoxy composites [37].

## 5. Conclusions

The present study deals with the utilization of wastes resources (i.e., carbon particles and Kevlar fabrics) to create hybrid composites with higher electrical properties coupled with high mechanical strength. For this purpose, the obtained activated carbon microparticles were ground to convert them into suitable nanofillers, whereas the Kevlar fabrics waste was used as a source to improve the mechanical strength of the composite. Moreover, the green resin was selected for a relatively easier bid-degradability of composites than those of conventional epoxy resins. At first, the conductive paste was made using different concentrations of fillers. The developed composite samples with 8 percent of carbon particles showed the highest electrical properties. In the second part of the study, the conductive paste was further used to develop the conductive composites. A similar trend was observed for the electrical conductivity and electromagnetic shielding effectiveness of composites, which were lower for 6 g of carbon fillers and highest for 8 g of carbon fillers. The mechanical performance of these conductive composites also increased with the said parameters. The composite sample with 8 g of carbon particles led to the lowest electrical resistivity of 89 Ωmm, EMI shielding (35.6 dB), and excellent mechanical performance. Furthermore, the joule heating response of the developed conductive composites was studied by measuring the change in temperature at the surface of the conductive composite samples while applying a voltage difference across the composite sample. A maximum temperature of 55 °C was obtained when the applied voltage was 10 V. Moreover, to estimate the durability and activity in service, the ageing performance (mechanical strength) of the developed composite samples was also analyzed. Composite samples with 2 g of carbon fillers had the lowest mechanical strength as compared to those with 8 g of carbon particles. It means the concentration of conductive fillers has a significant effect on the increase in mechanical properties

## Figures and Tables

**Table 1:** Design of experiments.

No of Composites Samples	Treatment	Sample ID	Concentrations of Carbon Particles (Grams)
1	Chemical treatment	CH1	2
2		CH2	4
3		CH3	6
4		CH4	8
5	untreated	C5	0

## Data Availability

The study did not report any data.
